# Sex and Age Differences in Clinicopathological Characteristics of Gastric Cancer

**DOI:** 10.3390/jcm14227894

**Published:** 2025-11-07

**Authors:** Claus Schildberg, Ulrike Weber, Nina Dietrich, Ute Seeland, René Mantke

**Affiliations:** 1 Department of General and Viszeral Surgery, Brandenburg Medical School Theodor Fontane, University Hospital Brandenburg, 14770 Brandenburg, Germany; 2 Faculty of Health Sciences Brandenburg, Brandenburg Medical School Theodor Fontane, 16816 Neuruppin, Germany; 3 Department of Internal Medicine, Section Sex- and Gender-Sensitive Medicine and Prevention, Otto-von-Guericke University Magdeburg, 39106 Magdeburg, Germany; ute.seeland@med.ovgu.de; 4 German Society of Gender-Specific Medicine, 14467 Potsdam, Germany

**Keywords:** gastric carcinoma, adenocarcinoma, real-world data, sex differences, age differences

## Abstract

**Background**: There is a lack of population-based and real-world data on sex and age differences in gastric cancer care. The aim of this study was to close this data gap and to analyze the sex and age differences in the clinicopathological characteristics of gastric adenocarcinoma. **Methods**: The analysis focused on patients diagnosed with gastric adenocarcinomas (ICD-10: C16.0–C16.9) documented in the cancer registry of the Federal State of Brandenburg from 2000 to 2020. The patient variables include sex, age at time of tumor diagnosis and the ECOG performance status. The tumor variables included location, grading, clinical TNM classification, clinical UICC stage and synchronous distant metastasis. **Results**: Of *n* = 8582 patients, 38% (*n* = 3263) were women. Compared with males, females had fewer adenocarcinomas located in the cardia (24.1% vs. 12.5%, *p* < 0.001), more signet ring cell carcinomas (13.2% vs. 22.8%, *p* < 0.001), more high-grade tumors (55.4% vs. 63.1%, *p* < 0.001) and a higher tumor cUICC stage (cUICC IV: 44.9% vs. 48.4%, *p* = 0.001). There was an interaction between sex and age modulating the differences between males and females. **Conclusions**: We were able to demonstrate several relevant prognostic differences in gastric cancer between men and women in terms of tumor location, stage, and metastases in a large patient cohort.

## 1. Introduction

According to recent statistics, both the incidence of and mortality from gastric cancer are the fifth highest worldwide [1]. Gastric cancer is more common in males than in females [1]. This difference in incidence between males and females is likely due to the difference in exposure to some risk factors, like Helicobacter pylori infection, family history of gastric cancer, dietary habits, smoking and alcohol [1]. However, these factors do not completely explain the different characteristics of gastric cancer between the sexes. Some studies have concluded that exposure to estrogen could reduce the risk of gastric cancer [2,3,4,5].

There is a lack of current population-based and real-world data on sex and age differences in gastric cancer care. Analyses of sex differences primarily originate from Asia or focus on older cohorts [6,7]. In the Netherlands, Kalff et al. [8] conducted a study in patients with gastric adenocarcinoma (*n* = 2072) registered in the Dutch Upper GI Cancer Audit between 2011 and 2016. In their study, patients without metastatic gastric cancer and with an elective surgery were included.

To close this sex and age data gap for patients, including those with UICC IV stage and without surgery, the aim of our study was to analyze the sex and age differences in clinicopathological features and staging in gastric adenocarcinoma using cancer registry data from the Federal State of Brandenburg from 2000 to 2020.

## 2. Methods

We conducted a cohort analysis according to STROBE (STrengthening the Reporting of OBservational studies in Epidemiology) [9] and SAGER (Sex and Gender Equity in Research) [10] guidelines for reporting observational studies with routinely collected data according to sex.

### 2.1. Study Design

The study was a retrospective observational study using routinely collected data from 2000 to 2020.

### 2.2. Data Source

The data was provided by the joint clinical–epidemiological Cancer Registry Brandenburg and Berlin gGmbH. This joint registry was established on 1 July 2016. In accordance with § 65c of the German Social Security Code V (SGB V), the primary objective of the registry is to record the occurrence, treatment, and course of malignant tumors, including their early stages, by inpatients and outpatients over the age of 18.

All doctors, dentists, and psychological psychotherapists who provide oncological services in Brandenburg or Berlin, as well as doctors not in contact with patients who provide services for treating doctors (e.g., pathologists, laboratory medicine), are obligated to report their findings in the registry.

Reporting is performed with the uniform oncological basic data set of the ADT (Working Group of German Tumor Centers)/GEKID (Society of Epidemiological Cancer Registries in Germany).

For documentation of primary tumors, the ICD-10-GM is used for diagnosis and location, the ICD-O-3 for morphology, and the UICC/AJCC TNM criteria of the respective TNM editions are used for classification of the extent of the primary tumor and metastases [11]. It should be noted that the TNM criteria for gastric cancer have been redefined as of 2010 [12], so a separate analysis of the TNM criteria data from 2000 to 2009 and from 2010 to 2020 seems reasonable.

Also, it should be noted that following the establishment of a joint cancer registry in 2016, a new reporting obligation for tumor diseases was introduced, one that had not previously existed in this form. Consequently, there has been a substantial increase in the number of cases for all tumor entities, including gastric adenocarcinoma, which is related to the aforementioned reporting obligation and, consequently, a more complete recording of tumor diseases.

### 2.3. Study Population

The analysis focused on patients diagnosed with gastric adenocarcinomas (ICD-10-GM: C16.0–C16.9) between 2000 and 2020 and residing in the Federal State of Brandenburg. The data status was 31 December 2020. In the period between 2000 and 2020, a total of 8582 patients residing in the state Brandenburg with newly diagnosed gastric adenocarcinoma were documented in the Cancer Registry and were included in the analysis in this study.

### 2.4. Variables

The patient variables of our analysis include sex, age at time of tumor diagnosis and ECOG performance status. As female sex hormones, i.e., estrogen, might have a protective effect reducing the risk and invasiveness of gastric adenocarcinoma, age was divided into three subgroups: <45 years, 45–60 years and older than 60 years. The tumor variables included location, grading, clinical TNM classification, clinical UICC stage and synchronous distant metastasis (synchronous: time between diagnosis of tumor and distant metastasis <= 3 months).

### 2.5. Statistical Methods

Age-standardized incidence and mortality rates (ESR, based on the old European standard) were calculated to show temporal trends. Patient and tumor characteristics were presented using absolute and relative frequencies for categorical variables and medians (IQRs) for continuous variables. For overall comparisons, χ^2^ tests, Fisher’s exact tests and Wilcoxon’s signed-rank tests were performed.

The study was exploratory, so no correction for multiple tests was performed. The significance level was locally defined and should be interpreted descriptively. Missing data were not imputed and were reported. The analysis was performed using R version 4.3.0.

## 3. Results

### 3.1. Sex and Age Differences in Patient Characteristics

**Figure 1:**
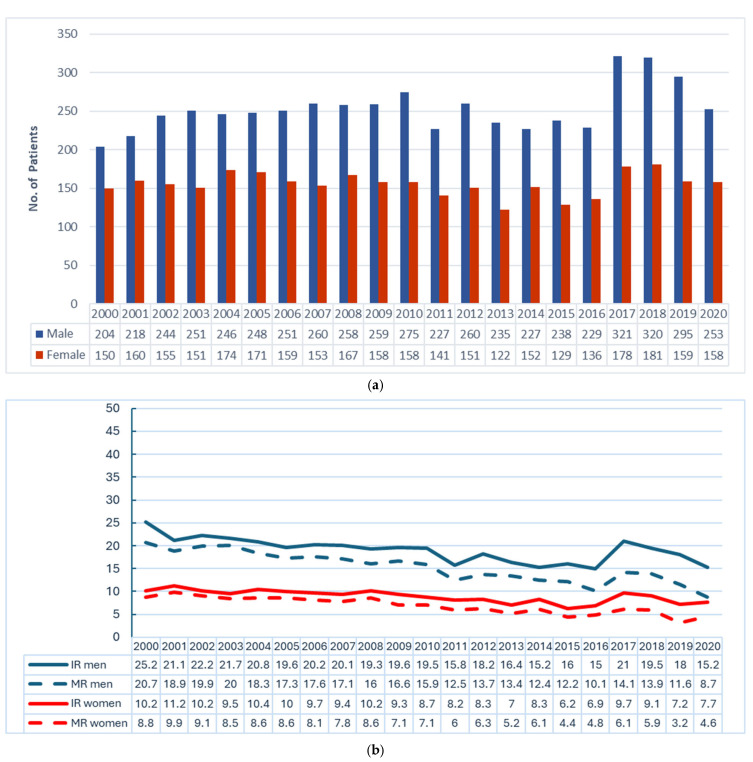
Gastric adenocarcinoma cases in the Federal State of Brandenburg, 2000–2020. Data: Cancer Registry Brandenburg–Berlin. (**a**) Number of patients by sex, *n* = 8582. (**b**) Age-standardized incidence (IR) and mortality rates (MRs) by sex; gastric adenocarcinoma cases in the Federal State of Brandenburg (*n* = 8582), 2000–2020, per 100.000 (European standard population 1976).

In the period from 2000 to 2020, a total of *n* = 8582 new cases of gastric adenocarcinoma were registered in people residing in the Federal State of Brandenburg. Of these patients, 62% patients (*n* = 5319) were men, and 38% (*n* = 3263) were women. Since the year 2000, there has been a consistent ratio of approximately two males to one female among the annual new cases of gastric cancer (Figure 1a,b). There was a continuous decline in the age-standardized incidence and mortality rates of gastric adenocarcinoma (Figure 1b) in both sexes.

At the time of diagnosis, females, with a median age of 73 years (IQR, 64–80 years), tended be older than males, with a median age of 70 years (IQR, 61–77 years) (*p* < 0.001) (Table 1). The prevalence of previous tumors was found to be lower among female patients (10.4%, *n* = 338) than among their male counterparts (12.7%, *n* = 674) (*p* = 0.011) (Table 1). The ECOG performance status was better among male patients than among females (Table 1). As expected, there was an increase in previous tumor prevalence and a decrease in EGOG status with older age (Table 1).

### 3.2. Sex and Age Differences in Tumor Characteristics and Distant Metastasis

In total, 23.9% of adenocarcinomas were localized in the body (*n* = 2048), 22.2% (*n* = 1913) in the antrum, and 19.7% in the cardia (*n* = 1692). The values for other, less frequent locations can be found in Table 2.

**Figure 2:**
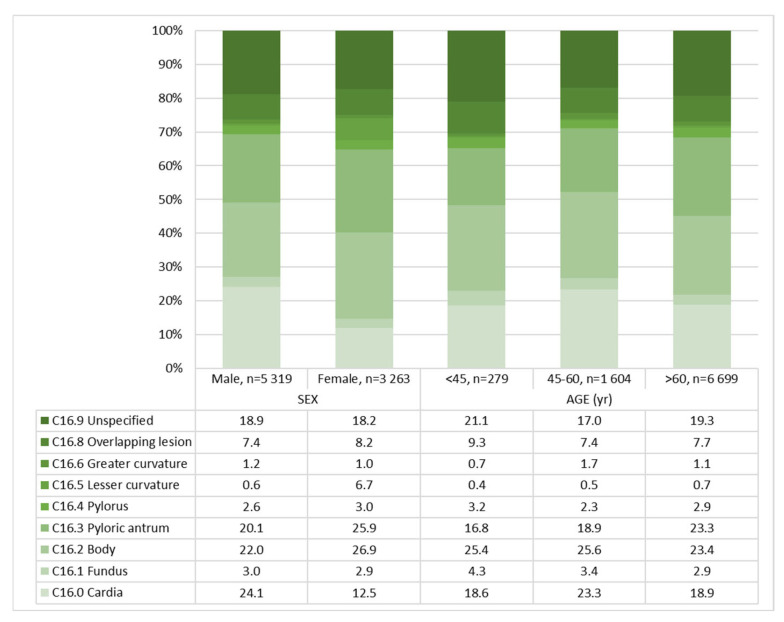
Tumor location—gastric adenocarcinoma in the Federal State of Brandenburg, 2000–2020, in % for sex and age, *n* = 8582. Data: Cancer Registry Brandenburg–Berlin.

**Figure 3:**
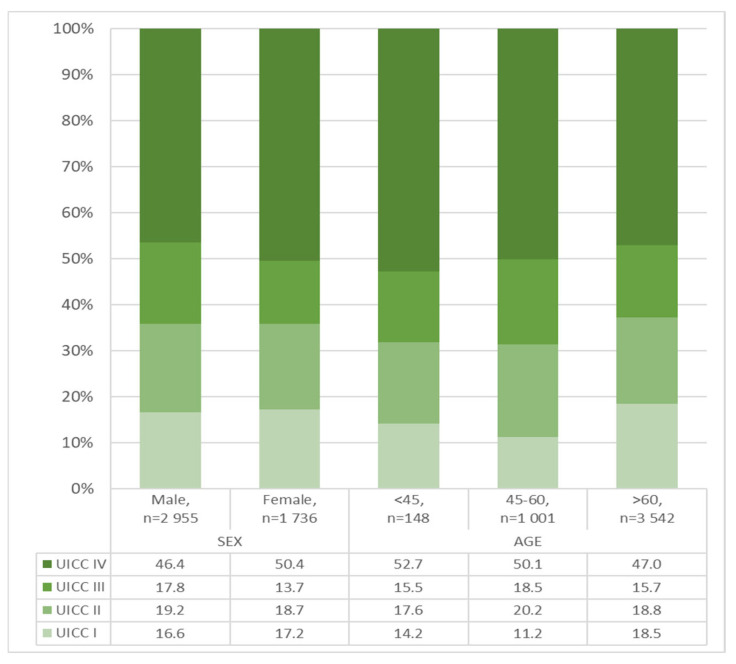
Clinical UICC stage—gastric adenocarcinoma in the Federal State of Brandenburg, 2000–2020, in % for sex and age, *n* = 4691, missing: *n* = 3891. Data: Cancer Registry Brandenburg–Berlin.

**Figure 4:**
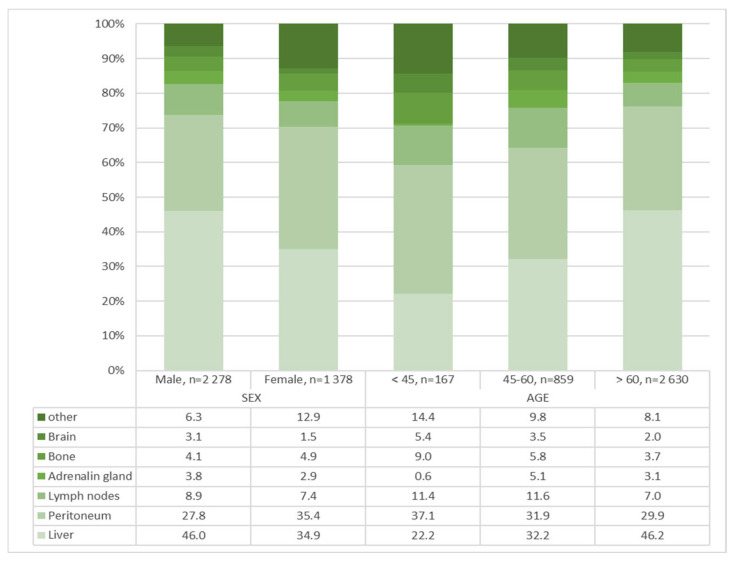
Synchronous distant metastases—gastric adenocarcinoma in the Federal State of Brandenburg, 2000–2020, by sex and age in %, *n* = 3656. Synchronous distant metastases: time between diagnosis of tumor and metastasis <= 3 months. Multiple metastases per patient possible. Data: Cancer registry Brandenburg–Berlin.

Compared with males, females had fewer adenocarcinomas located in the cardia (24.1% males vs. 12.5% females, *p* < 0.001) (Figure 2, Table 2), more signet ring cell carcinomas (13.2% males vs. 22.8% females, *p* < 0.001) (Table 2), more high-grade tumors (55.4% males vs. 63.1% females, *p* < 0.001) (Table 2), a higher tumor cUICC stage (cUICC IV: 46.4% males vs. 50.4% females, *p* = 0.001) (Figure 3, Table 2) and fewer synchronous distant metastases in the liver (46.0% males vs. 34.9% females, *p* < 0.001) (Figure 4, Table 3).

With respect to age, the highest proportion of signet ring cell carcinomas (32.3%, *n* = 90) was observed in the under-45 age group (*n* = 279). A slightly higher proportion in this age group (52.7%, *n* = 78) exhibited UICC stage IV disease, and a notably higher proportion (71.3%, *n*= 186) displayed poorly differentiated adenocarcinomas (Table 2 and Figure 3). Figure 4 and Table 3 provide an overview of the age dependence of the synchronous distant metastases that occurred.

Regarding the TNM classification, there were minimal differences between males and females in the cT category, but females tended to have more distant metastases (cM1 category) (Table 4a,b).

### 3.3. Sex Differences in Patient and Tumor Characteristics by Age Group

Regarding the ratio of males to females across the three distinct age groups, there was a notable observation in the youngest age group (age < 45 years). In this age group (*n* = 279), the number of females (*n* = 134, 48.2%) nearly equals that of males (*n* = 145, 51.2%), indicating a balanced gender distribution within this group. In relation to age distribution, the proportion of women under 45 years of age is higher than proportion of men in this age group (4.1% females vs. 2.7% males, *p* < 0.001) (Table 1).

Female patients have a lower prevalence of cardia-located gastric carcinoma across all age groups. This is particularly pronounced in the youngest age group < 45 years (28.3% males vs. 8.2% females, *p* < 0.001). There are also sex differences in the histology subtype in all age groups. So, females are more frequently diagnosed with diffuse and signet cell carcinoma-type tumors (Table 5).

Female gastric cancer patients have a poorer tumor differentiation and higher cUICC stage across all age groups compared to males (Table 5). The inter-sex differences in all tumor characteristics are less pronounced in the oldest age group (>60 years).

Distant metastases in the peritoneum occur more frequently in females than in males in all age groups. This effect is particularly evident in the youngest age group (30.4% males vs. 43.2% females, *p* < 0.072). In all age groups, the frequency of distant liver metastases is lower in female patients. In oldest age group, this sex difference is less obvious (49.8% males vs. 40.4% females, *p* < 0.001) (Table 6).

## 4. Discussion

The aim of our study was to analyze the sex and age differences in clinicopathological features and staging in gastric adenocarcinoma patients using cancer registry data from the Federal State of Brandenburg from 2000 to 2020. In contrast to other real-word studies, we described all patients, including those with a UICC IV staging status and no surgery.

In general, the incidence of gastric cancer is lower in females than in males [1], which was also found in our study. The reason for this difference is still unclear, since it cannot be fully explained based on differences in exposure to known risk factors. This also applies to carcinomas of the gastroesophageal junction [1].

A higher incidence of gastric cancer in younger females was found in our study. According to Luan et al. [13], it is suggested that, on the one hand, the use of exogenous hormones could reduce the risk of gastric cancer. On the other hand, higher estrogen levels and a higher proportion of estrogen receptor-positive cells have been found in younger females, and these might be responsible for the higher risk for gastric cancer in young females.

With regard to the assessment of a patient’s general condition, and thus their suitability for, e.g., systemic therapies, the results of our study indicated a poorer ECOG performance status in females.

In line with Kalff et al. [8] and Luan et al. [13], females in our study had fewer adenocarcinomas located in the cardia, more signet ring cell carcinomas, more high-grade tumors, a higher tumor cUICC stage and more synchronous distant metastases in the peritoneum. In particular, younger female gastric cancer patients were more often diagnosed with poorly differentiated and diffuse and signet cell carcinoma. It is known that these tumors have unfavorable prognostic characteristics and poorer survival rates.

Moreover, our study showed an interaction between sex and age modulating the differences between males and females, especially in the age group over 60 years. This finding could indicate that the clinicopathology of gastric cancer in older women becomes similar to that of men due to the decreasing hormonal influence.

Analyses of registry data are subject to certain limitations. Despite obligations, not all patients diagnosed with gastric cancer are documented. However, the Robert Koch Institute asserts that a 90% registration rate can be assumed for the Brandenburg Berlin Cancer Registry from 2008 onwards. Furthermore, there are no data concerning co-morbidities. There is also no information available on the lifestyle (risk) factors relevant to gastric cancer. According to Loew et al. [14], a further limitation is the incomplete reporting of clinical tumor stages in the cancer registry, with approximately 30% of missing data. However, there is no reason to suspect that they are not missing at random with regard to sex and age. Another limitation was that we could not provide information on Helicobacter pylori status. This is important because there is evidence for the involvement of estrogen levels in the carcinogenesis of Helicobacter-positive carcinomas [15].

In summary, there are sex differences in the clinicopathological characteristics of gastric adenocarcinoma in the Federal State of Brandenburg which are moderated by increasing age. In the next step of our project, homogeneous groups are to be built by statistical methods in order to evaluate whether females and males with similar clinicopathology characteristics undergo different surgical and/or systemic therapies.

In the future, it would also be very important to investigate the distinctions between pre- and postmenopausal females. This appears to influence both the clinical manifestation of gastric cancer in females and its carcinogenesis. This should also be considered with regard to survival, as women have different survival rates compared to men. There is also evidence that this is related to pre- and postmenopausal estrogen levels [16,17].

## Figures and Tables

**Table 1:** Patient characteristics by sex and by age—gastric adenocarcinoma in the Federal State of Brandenburg, 2000–2020, *n* = 8582 patients. Data: Cancer Registry Brandenburg–Berlin.

Characteristic	Total (*n* = 8582)	By Sex			By Age Group (Year)			
		Males (*n* = 5319)	Females (*n* = 3263)	*p*-Value	<45 (*n* = 279)	45–60 (*n* = 1604)	>60 (*n* = 6699)	*p* Value
	***no.*** **(%)**	***no.*** **(%)**			***no.*** **(%)**			
**Age**				<0.001				<0.001
Median (IQR)	71.0 (62.0–78.0)	70.0 (61.0–77.0)	73.0 (64.0–80.0)		40.0 (36.0–43.0)	55.0 (50.0–58.0)	74.0 (68.0–80.0)	
**Age Group (Year)**				<0.001				
<45	279 (3.3)	145 (2.7)	134 (4.1)		-	-	-	
45–60	1604 (18.7)	1100 (20.7)	504 (15.4)		-	-	-	
>60	6699 (78.0)	4074 (76.6)	2625 (80.5)		-	-	-	
**Previous Tumors**				0.011				<0.001
yes	1012 (11.8)	674 (12.7)	338 (10.4)		6 (2.2)	99 (6.2)	907 (13.5)	
no	7570 (88.2)	4645 (87.3)	2925 (89.6)		273 (97.8)	1505 (93.8)	5792 (86.5)	
**ECOG**				<0.001				<0.001
0	1144 (22.9)	752 (24.5)	392 (20.2)		59 (38.1)	345 (36.8)	740 (18.9)	
1	1614 (32.3)	1007 (32.9)	607 (31.3)		63 (40.6)	318 (33.9)	1233 (31.5)	
2	1494 (29.9)	905 (29.5)	589 (30.4)		26 (16.8)	225 (24.0)	1243 (31.8)	
3	599 (12.0)	327 (10.7)	272 (14.0)		6 (3.9)	41 (4.4)	552 (14.1)	
4	153 (3.1)	73 (2.4)	80 (4.1)		1 (0.6)	8 (0.9)	144 (3.7)	
missing	3578	2255	1323		124	667	2787	

ECOG categories: 0 = fully active, able to carry on all pre-disease performance without restriction; 1 = restricted in physically strenuous activity but ambulatory and able to carry out work of a light or sedentary nature, e.g., light house work, office work; 2 = ambulatory and capable of all self-care but unable to carry out any work activities; up and about more than 50% of waking hours; 3 = capable of only limited self-care; confined to bed or chair more than 50% of waking hours; 4 = completely disabled; cannot carry on any self-care; totally confined to bed or chair.

**Table 2:** Tumor characteristics by sex and by age—gastric adenocarcinoma in the Federal State of Brandenburg, 2000–2020, *n* = 8582 patients. Data: Cancer Registry Brandenburg–Berlin.

Characteristic	Total (*n* = 8582)	By Sex			By Age Group (Year)			
		Males (*n* = 5319)	Females (*n* = 3263)	*p*-Value	<45 (*n* = 279)	45–60 (*n* = 1604)	>60 (*n* = 6699)	*p* Value
	***no.*** **(%)**	***no.*** **(%)**			***no.*** **(%)**			
**Location**				<0.001				<0.001
C16.0 Cardia	1692 (19.7)	1284 (24.1)	408 (12.5)		52 (18.6)	374 (23.3)	1266 (18.9)	
C16.1 Fundus	257 (3.0)	162 (3.0)	95 (2.9)		12 (4.3)	54 (3.4)	191 (2.9)	
C16.2 Body	2048 (23.9)	1170 (22.0)	878 (26.9)		71 (25.4)	411 (25.6)	1566 (23.4)	
C16.3 Pyloric antrum	1913 (22.2)	1069 (20.1)	844 (25.9)		47 (16.8)	303 (18.9)	1563 (23.3)	
C16.4 Pylorus	238 (2.8)	139 (2.6)	99 (3.0)		9 (3.2)	37 (2.3)	192 (2.9)	
C16.5 Lesser curvature	55 (0.6)	33 (0.6)	22 (6.7)		1 (0.4)	8 (0.5)	46 (0.7)	
C16.6 Greater curvature	100 (1.2)	66 (1.2)	34 (1.0)		2 (0.7)	27 (1.7)	71 (1.1)	
C16.8 Overlapping lesion	658 (7.7)	392 (7.4)	266 (8.2)		26 (9.3)	118 (7.4)	514 (7.7)	
C16.9 Unspecified	1621 (18.9)	1004 (18.9)	616 (18.2)		59 (21.1)	272 (17.0)	1290 (19.3)	
**Histology**				<0.001				<0.001
Adenocarcinoma, n. specified	4041 (47.1)	2677 (50.3)	1364 (41.8)		81 (29.0)	681 (42.5)	3279 (48.9)	
Intestinal	1547 (18.0)	1028 (19.3)	519 (15.9)		31 (11.1)	236 (14.7)	1280 (19.1)	
Diffuse	845 (9.8)	456 (8.6)	389 (11.9)		53 (19.0)	200 (12.5)	592 (8.8)	
Tubular	384 (4.5)	257 (4.8)	127 (3.9)		9 (3.2)	36 (2.2)	339 (5.1)	
Papillary	26 (0.3)	18 (0.3)	8 (0.2)		0 (0.0)	6 (0.4)	20 (0.3)	
Signet cell carcinoma	1440 (16.8)	700 (13.2)	744 (22.8)		90 (32.3)	387 (24.1)	967 (14.4)	
Mixed adenocarcinoma	53 (0.6)	32 (0.6)	21 (0.6)		0 (0.0)	11 (0.7)	42 (0.6)	
Other	242 (2.8)	151 (2.8)	91 (2.8)		15 (5.4)	47 (2.9)	180 (2.7)	
**Grading**				<0.001				<0.001
G1 well differentiated	415 (5.2)	280 (5.6)	135 (4.4)		4 (1.5)	50 (3.4)	361 (5.8)	
G2 moderately differentiated	2461 (30.7)	1666 (33.5)	795 (26.2)		43 (16.5)	345 (23.2)	2073 (33.1)	
G3 poorly differentiated	4671 (58.3)	2757 (55.4)	1914 (63.1)		186 (71.3)	987 (66.4)	3498 (55.8)	
G4 undifferentiated	134 (1.7)	66 (1.3)	68 (2.2)		10 (3.8)	37 (2.5)	87 (1.4)	
GX cannot be assessed	330 (4.1)	208 (4.2)	122 (4.0)		18 (6.9)	67 (4.5)	245 (3.9)	
missing	571	342	229		18	118	435	
**cUICC**				0.001				<0.001
I	789 (16.8)	490 (16.6)	299 (17.2)		21 (14.2)	112 (11.2)	656 (18.5)	
II	893 (19.0)	568 (19.2)	325 (18.7)		26 (17.6)	202 (20.2)	665 (18.8)	
III	763 (16.3)	526 (17.8)	237 (13.7)		23 (15.5)	185 (18.5)	555 (15.7)	
IV	2246 (47.9)	1371 (46.4)	875 (50.4)		78 (52.7)	502 (50.1)	1666 (47.0)	
Missing	3891	2364	1527		131	603	3157	

**Table 3:** Synchronous distant metastases by sex and by age—gastric adenocarcinoma in the Federal State of Brandenburg, 2000–2020, *n* = 3656 patients. Data: Cancer Registry Brandenburg–Berlin.

Characteristic	Total (*n* = 3656)	By Sex			By Age Group (Year)			
		Males (*n* = 2278)	Females (*n* = 1378)	*p*-Value	<45 (*n* = 167)	45–60 (*n* = 859)	>60 (*n* = 2630)	*p* Value
	***no.*** **(%)**	***no.*** **(%)**			***no.*** **(%)**			
**Distant metastasis**				<0.001				<0.001
Liver	1530 (41.8)	1049 (46.0)	481 (34.9)		37 (22.2)	277 (32.2)	1216 (46.2)	
Peritoneum	1122 (30.7)	634 (27.8)	488 (35.4)		62 (37.1)	274 (31.9)	786 (29.9)	
Lymph node	304 (8.3)	202 (8.9)	102 (7.4		19 (11.4)	100 (11.6)	185 (7.0)	
Bone	162 (4.4)	94 (4.1)	68 (4.9)		15 (9.0)	50 (5.8)	97 (3.7)	
Adrenal gland	126 (3.4)	86 (3.8)	40 (2.9)		1 (0.6)	44 (5.1)	81 (3.1)	
Brain	91 (2.5)	70 (3.1)	21 (1.5)		9 (5.4)	30 (3.5)	52 (2.0)	
Other	321 (8.8)	143 (6.3)	178 (12.9)		24 (14.4)	84 (9.8)	213 (8.1)	

Synchronous distant metastases: Time between diagnosis of tumor and metastasis <= 3 months. Multiple metastases per patient possible.

**Table 4:** (**a**) cTNM classification (4., 5. and 6. edition) by sex and by age—gastric adenocarcinoma in the Federal State of Brandenburg, 2000–2009, *n* = 4037 patients. Data: Cancer Registry Brandenburg–Berlin. (**b**) cTNM classification (7. and 8. edition) by sex and by age—gastric adenocarcinoma in the Federal State of Brandenburg, 2010–2020, *n* = 4545 patients. Data: Cancer Registry Brandenburg-Berlin.

(a)								
Characteristic	Total (*n* = 4037)	By Sex			By Age Group (Year)			
		Males (*n* = 2439)	Females (*n* = 1598)	*p*-Value	<45 (*n* = 174)	45–60 (*n* = 755)	>60 (*n* = 3108)	*p* Value
	***no.*** **(%)**	***no.*** **(%)**			***no.*** **(%)**			
**cT Category**				0.304				0.156
T0: No evidence of tumor	1 (0.1)	0 (0.0)	1 (0.2)		0 (0)	1 (0.3)	0 (0)	
Tis: In situ	3 (0.2)	2 (0.2)	1 (0.2)		0 (0.)	1 (0.3)	2 (0.2)	
T1: Lamina propria or submucosa	183 (11.3)	110 (11.2)	73 (11.4)		7 (10.0)	23 (6.8)	153 (12.6)	
T2: Muscularis propria or subserosa	242 (14.9)	146 (14.9)	96 (14.9)		8 (11.4)	52 (15.3)	182 (15.0)	
T3: Serosa	466 (28.7)	301 (30.7)	165 (25.7)		22 (31.4)	106 (31.2)	338 (27.8)	
T4: Adjacent structures	334 (20.6)	190 (19.4)	144 (22.4)		11 (15.7)	75 (22.1)	248 (20.4)	
TX: Cannot be assessed	395 (24.3)	232 (23.6)	163 (25.3)		22 (31.4)	82 (24.1)	291 (24.0)	
*Missing*	2413	1458	955		104	415	1894	
**cN Category**				0.2				0.019
N0: No met. in regional lymph nodes	307 (18.9)	195 (19.9)	112 (17.4)		11 (15.9)	60 (17.6)	236 (19.4)	
N1: 1 to 6 regional lymph nodes	407 (25.1)	255 (26.0)	152 (23.6)		16 (23.2)	100 (29.3)	291 (24.0)	
N2: 7 to 15 regional lymph nodes	170 (10.5)	106 (10.8)	64 (10.0)		4 (5.8)	40 (11.7)	126 (10.4)	
N3: More than 15 regional lymph nodes	61 (3.8)	31 (3.2)	30 (4.7)		7 (10.1)	16 (4.7)	38 (3.1)	
NX: Cannot be assessed	679 (41.8)	394 (40.2)	285 (44.3)		31 (44.9)	125 (36.7)	523 (43.1)	
*Missing*	2413	1458	955		105	414	1894	
**cM Category**				0.002				<0.015
M0: No distant metastasis	568 (34.6)	375 (37.9)	193 (29.6)		20 (27.8)	133 (38.8)	415 (33.8)	
M1: Distant metastasis	843 (51.3)	487 (49.2)	356 (54.6)		44 (61.1)	178 (51.9)	621 (50.6)	
MX: Cannot be assessed	231 (14.1)	128 (12.9)	103 (15.8)		8 (11.1)	32. (9.3)	191 (15.6)	
*Missing*	2395	1449	946		102	412	1881	
(**b**)								
**Characteristic**	**Total** **(*n* = 4545)**	**By Sex**			**By Age Group (Year)**			
		**Males** **(*n* = 2880)**	**Females** **(*n* = 1665)**	***p*** **-Value**	**<45** **(*n* = 105)**	**45–60** **(*n* = 849)**	**>60** **(*n* = 3591)**	***p*** **Value**
	***no.*** **(%)**	***no.*** **(%)**			***no.*** **(%)**			
**cT Category**				0.9				0.010
T0: No evidence of tumor	0 (0)	0 (0)	0 (0)		0 (0)	0 (0)	0 (0)	
Tis: In situ	8 (0.3)	5 (0.2)	3 (0.3)		0 (0)	0 (0)	8 (0.3)	
T1: Lamina propria or submucosa	320 (10.1)	205 (10.1)	115 (10.1)		6 (7.3)	38 (5.7)	276 (11.5)	
T2: Muscularis propria or subserosa	427 (13.5)	271 (13.4)	156 (13.8)		9 (11.0)	97 (14.5)	321 (13.3)	
T3: Serosa	1228 (38.9)	802 (39.6)	426 (37.6)		37 (45.1)	278 (41.4)	913 (37.9)	
T4: Adjacent structures	619 (19.6)	393 (19.4)	226 (19.9)		17 (20.7)	151 (22.5)	451 (18.7)	
TX: Cannot be assessed	557 (17.6)	349 (17.2)	208 (18.3)		13 (15.9)	107 (15.9)	437 (18.2)	
*Missing*	1386	855	531		23	178	1185	
**cN Category**				0.014				<0.001
N0: No met. in regional lymph nodes	865 (27.7)	554 (27.6)	311 (27.8)		21 (26.6)	139 (20.9)	705 (29.6)	
N1: 1 to 6 regional lymph nodes	1086 (34.8)	714 (35.6)	372 (33.3)		30 (38.0)	272 (41.0)	784 (32.9)	
N2: 7 to 15 regional lymph nodes	340 (10.9)	235 (11.7)	105 (9.4)		10 (12.7)	79 (11.9)	251 (10.5)	
N3: More than 15 regional lymph nodes	187 (6.0)	122 (6.1)	65 (5.8)		5 (6.3)	44(6.6)	138(5.8)	
NX: Cannot be assessed	646 (20.7)	381 (19.0)	265 (23.7)		13 (16.5)	130 (19.6)	503 (21.1)	
*Missing*	1421	874	547		26	185	1210	
**cM Category**				0.5				0.663
M0: No distant metastasis	1782 (55.9)	1158.0 (56.5)	624 (54.6)		47 (56.6)	358 (53.2)	1377 (56.6)	
M1: Distant metastasis	1278 (40.1)	806 (39.4)	472 (41.3)		33 (39.8)	287 (42.6)	958 (39.4)	
MX: Cannot be assessed	130 (4.1)	84 (4.1)	46 (4.0)		3 (3.6)	28 (4.2)	99 (4.1)	
*Missing*	1355	832	523		22	176	1157	

**Table 5:** Tumor characteristics by sex by age group—gastric adenocarcinoma in the Federal State of Brandenburg, 2000–2020, *n* = 8528 patients. Data: Cancer Registry Brandenburg–Berlin.

Characteristic	Age < 45 Years			Age 45 to 59 Years			Age 60 Years and Older		
	Males (*n* = 145)	Females (*n* = 134)	*p*-Value	Males (*n* = 1100)	Females (*n* = 504)	*p*-Value	Males (*n* = 4074)	Females (*n* = 2625)	*p*-Value
**Location**			<0.001			<0.001			<0.001
C16 Cardia	41 (28.3)	11 (8.2)		317 (28.8)	57 (11.3)		926 (22.7)	340 (13.0)	
C16.1 Fundus	6 (4.1)	6 (4.5)		38 (3.5)	16 (3.2)		118 (2.9)	73 (2.8)	
C16.2 Body	33 (22.8)	38 (28.4)		241 (21.9)	170 (33.7)		896 (22)	670 (25.5)	
C16.3 Pyloric antrum	22 (15.2)	25 (18.7)		200 (18.2)	103 (20.4)		847 (20.8)	716 (27.3)	
C16.4 Pylorus	6 (4.1)	3 (2.2)		29 (2.6)	8 (1.6)		104 (2.6)	88 (3.4)	
C16.5 Lesser curvature	0 (0)	1 (0.7)		5 (0.5)	3 (0.6)		28 (0.7)	18 (0.7)	
C16.6 Greater curvature	0 (0)	2 (1.5)		21 (1.9)	6 (1.2)		45 (1.1)	26 (1.0)	
C16.8 Overlapping lesion	11 (7.6)	15 (11.2)		76 (6.9)	42 (8.3)		305 (7.5)	209 (8.0)	
C16.9 Unspecified	26 (17.9)	33 (24.6)		173 (15.7)	99 (19.6)		805 (19.8)	485 (18.5)	
**Histology**			0.018			<0.001			<0.001
Adenocarcinoma, n. specified	45 (31)	36 (26.9)		506 (46)	175 (34.7)		2126 (52.2)	1153 (43.9)	
Intestinal	21 (14.5)	10 (7.5)		194 (17.6)	42 (8.3)		813 (20.0)	467 (17.8)	
Diffuse	23 (15.9)	30 (22.4)		120 (10.9)	80 (15.9)		313 (7.7)	279 (10.6)	
Tubular	7 (4.8)	2 (1.5)		31 (2.8)	5 (1.0)		219 (5.4)	120 (4.6)	
Papillary	0 (0)	0 (0)		5 (0.5)	1 (0.2)		13 (0.3)	7 (0.3)	
Signet cell carcinoma	38 (26.2)	52 (38.8)		205 (18.6)	182 (36.1)		457 (11.2)	510 (19.4)	
Mixed adenocarcinoma	0 (0)	0 (0)		6 (0.5)	5 (1.0)		26 (0.6)	16 (0.6)	
Other	11 (7.6)	4 (3.0)		33 (3.0)	14 (2.8)		107 (2.6)	73 (2.8)	
**Grading**			<0.001			<0.001			<0.001
G1 well differentiated	4 (2.9)	0 (0)		42 (4.1)	8 (1.7)		234 (6.1)	127 (5.2)	
G2 moderately differentiated	33 (24.1)	10 (8.1)		280 (27.3)	65 (14.1)		1353 (35.5)	720 (29.4)	
G3 poorly differentiated	84 (61.3)	102 (82.3)		635 (62.0)	352 (76.2)		2038 (53.4)	1460 (59.6)	
G4 undifferentiated	5 (3.6)	5 (4.0)		21 (2.1)	16 (3.5)		40 (1.0)	47 (1.9)	
GX cannot be assessed	11 (8.0)	7 (5.6)		46 (4.5)	21 (4.5)		151 (4.0)	94 (3.8)	
*Missing*	8	10		76	42		258	177	
**cUICC**			0.2			0.2			0.033
I	9 (11.8)	12 (16.7)		77 (11.2)	35 (11.2)		404 (18.4)	252 (18.6)	
II	14 (18.4)	12 (16.7)		140 (20.3)	62 (19.9)		414 (18.9)	251 (18.6)	
III	16 (21.1)	7 (9.7)		138 (20.0)	47 (15.1)		372 (17.0)	183 (13.5)	
IV	37 (48.7)	41 (56.9)		334 (48.5)	168 (53.8)		1000 (45.7)	666 (49.3)	
*Missing*	69	62		411	192		1884	1273	

**Table 6:** Distant metastasis by sex by age group—gastric adenocarcinoma in the Federal State of Brandenburg, 2000–2020, *n* = 3656 patients. Data: Cancer Registry Brandenburg-Berlin.

Characteristic	Age < 45 Years			Age 45 to 59 Years			Age 60 Years and Older		
	Males (*n* = 79)	Females (*n* = 88)	*p*-Value	Males (*n* = 576)	Females (*n* = 283)	*p*-Value	Males (*n* = 1623)	Females (*n* = 1007)	*p*-Value
	***no.*** **(%)**			***no.*** **(%)**			***no.*** **(%)**		
**Distant Metastasis**			0.072			<0.001			<0.001
Liver	20 (25.3)	17 (19.3)		220 (38.2)	57 (20.1)		809 (49.8)	407 (40.4)	
Peritoneum	24 (30.4)	38 (43.2)		171 (29.7)	103 (36.4)		439 (27.0)	347 (34.5)	
Lymph node	14 (17.7)	5 (5.7)		70 (12.2)	30 (10.6)		118 (7.3)	67 (6.7)	
Bone	7 (8.9)	8 (9.1)		28 (4.9)	22 (7.8)		59 (3.6)	38 (3.8)	
Adrenal gland	1 (1.3)	0 (0.0)		31 (5.4)	13 (4.6)		54 (3.3)	27 (2.7)	
Brain	5 (6.3)	4 (4.5)		21 (3.6)	9 (3.2)		44 (2.7)	8 (0.8)	
Other	8 10.1)	16 (18.2)		35 (6.1)	49 (17.3)		100 (6.2)	113 (11.2)	

## Data Availability

The original contributions presented in this study are included in the article. Further inquiries can be directed to the corresponding author.
